# Morphological and molecular characterization of freshwater prawn of genus *Macrobrachium* in the coastal area of Cameroon

**DOI:** 10.1002/ece3.5854

**Published:** 2019-12-08

**Authors:** Judith G. Makombu, Francesca Stomeo, Pius M. Oben, Eldridge Tilly, Opiyo O. Stephen, Benedicta O. Oben, Evans K. Cheruiyot, Getinet Mekuriaw Tarekegn, Paul Zango, Atem E. Egbe, Abigail Ndagyong, Eric Mialhe, Jules R. Ngueguim, Fidalis D. N. Mujibi

**Affiliations:** ^1^ Department of Fisheries and Aquatic Resources Management Faculty of Agriculture and Veterinary Medicine University of Buea Buea Cameroon; ^2^ Biosciences Eastern and Central Africa – International Livestock Research Institute (BecA‐ILRI) Hub Nairobi Kenya; ^3^ Molecular and Cellular Imaging Center–Columbus The Ohio State University Columbus OH USA; ^4^ University of the Sacred Heart Gulu Gulu Uganda; ^5^ USOMI Limited Nairobi Kenya; ^6^ School of Applied Systems Biology La Trobe University Bundoora Australia; ^7^ Department of Animal Breeding and Genetics Swedish University of Agricultural Sciences Uppsala Sweden; ^8^ Department of Animal Production and Technology Bahir Dar University Bahir Dar Ethiopia; ^9^ Institute of Fisheries and Aquatic Sciences Yabassi Cameroon; ^10^ Concepto Azul Cdlavernaza Norte Guayaquil Ecuador; ^11^ Institut de Recherche Agricole pour le Développement Limbe Cameroon; ^12^ Present address: European Molecular Biology Laboratory (EMBL) Heidelberg Germany

**Keywords:** Cameroon, DArT markers, freshwater prawn, Konan key, *Macrobrachium*, morphological and molecular characterization

## Abstract

*Macrobrachium* (Bate, 1868) is a large and cosmopolitan crustacean genus of high economic importance worldwide. We investigated the morphological and molecular identification of freshwater prawns of the genus *Macrobrachium* in South, South West, and Littoral regions of Cameroon. A total of 1,566 specimens were examined morphologically using a key described by Konan (Diversité morphologique et génétique des crevettes des genres Atya Leach, 1816 et Macrobrachium Bate, 1868 de Côte d'Ivoire, 2009, Université d'Abobo Adjamé, Côte d'Ivoire), leading to the identification of seven species of *Macrobrachium*: *M. vollenhovenii* (Herklots, 1857); *M. macrobrachion* (Herklots, 1851); *M. sollaudii* (De Man, 1912); *M. dux* (Lenz, 1910); *M. chevalieri* (Roux, 1935); *M. felicinum* (Holthuis, 1949); and an undescribed *Macrobrachium* species *M*.* *sp. To validate the genetic basis of the identified species, 94 individuals representing the species were selected and subjected to genetic characterization using 1,814 DArT markers. The admixture analysis revealed four groups: *M. vollenhovenii* and *M. macrobrachion*; *M. chevalieri*; *M. felicinum* and *M*.* *sp; and *M. dux* and *M. sollaudii*. But, the principal component analysis (PCA) separated *M*.* *sp and *M. felicinum* to create additional group (i.e., five groups). Based on these findings, *M. vollenhovenii* and *M. macrobrachion* may be conspecific, as well as *M. dux* and *M. sollaudii*, while *M. felicinum* and *M*.* *sp seems to be different species, suggesting a potential conflict between the morphological identification key and the genetic basis underlying speciation and species allocation for *Macrobrachium*. These results are valuable in informing breeding design and genetic resource conservation programs for *Macrobrachium* in Africa.

## Graphical abstract

*Macrobrachium* is a large crustacean genus of high economic importance. Morphological characterization and molecular characterization of prawns of this genus were undertaken in the three regions of the coastal area Cameroon. Seven species were identified morphologically: *M. vollenhovenii*; *M. macrobrachion*; *M. sollaudii*; *M. dux; M. chevalieri*; *M. felicinum*; and an undescribed *Macrobrachium* species *M*.* *sp, while genetic structure using 1,814 DArT markers showed close similarity between *M. vollenhovenii* and *M. macrobrachion*, and *M. dux* and *M. sollaudii*.

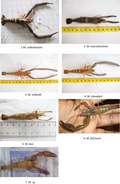

## 1 INTRODUCTION

The freshwater prawns of genus *Macrobrachium* (Crustacea, Decapoda, and Palaemonidea) constitute one of the most diverse, abundant, and widespread crustacean genera (Murphy & Austin, 2005). The species of this genus are distributed throughout the tropical and subtropical zones of the world (Fossati, Mosseron, & Keith, 2002; Holthuis, 1980; March, Pringle, Townsend, & Wilson, 2002). Various studies have identified approximately 240 species of *Macrobrachium* (Chen, Tsai, & Tzeng, 2009; De Grave & Fransen, 2011; Holthuis & Ng, 2010; Wowor et al., 2009). Although the majority of *Macrobrachium* species inhabit freshwaters, some are entirely restricted to estuaries and many require brackish water during larval development (New, 2002).

In West Africa, *Macrobrachium* species can be found throughout the region and play an important role in domestic fishery resources (Etim & Sankare, 1998; Nwosu & Wolfi, 2006). They are commercially important and sustain viable artisanal fisheries in some rivers and estuaries within the region, while also providing direct and secondary employment (Marioghae, 1990; Okogwu, Ajuogu, & Nwani, 2010). However, the species are poorly known in the region. Monod (1980) developed a *Macrobrachium* characterization key, which when applied to West Africa resulted in the identification of 10 species of *Macrobrachium*: *M. vollenhovenii* (Herklots, 1857), *M. macrobrachion* (Herklots, 1851), *M. chevalieri* (Roux, 1935), *M. dux* (Lenz, 1910), *M. felicinum* (Holthuis, 1949), *M. raridens* (Hilgendorf, 1893), *M. thysi* (Powell, 1980), *M. equidens* (Dana, 1852), *M. zariquieyi* (Holthius, 1949), and *M. sollaudii* (De Man, 1912), of which four are found in Cameroon: *M. vollenhovenii*, *M. macrobrachion*, *M. chevalieri*, and *M. sollaudii* (Monod, 1966, 1980; Powell, 1980). However, Monod (1980) cautioned that the use of his key is limited to adult males only.

Taking into consideration both sex and size of the prawn, Konan (2009) developed a new key for identification of West Africa *Macrobrachium*. Using the newly developed key, Makombu et al. (2015) described a tentative range of the biodiversity of *Macrobrachium* in the South region and increased the number of known species in Cameroon from four (Monod, 1980) to six (Makombu et al., 2015). Other studies also pointed out the higher species richness of Cameroon *Macrobrachium* (Doume, Toguyeni, & Yao, 2013; Tchakonté et al., 2014). However, these recent studies in Cameroon have not covered the whole coastal area, which encompasses three regions namely South, South West, and Littoral regions. With the increasing threat of the quality of fresh and brackish water of the coastal area of Cameroon (E & D, 2009; Folack, 1995) that can affect species integrity, information on the genetic diversity of *Macrobrachium* in the whole coastal region is urgently needed to implement a management plan.

Application of species identification keys relies heavily on distinct morphological features unique to each species. However, due to a restricted number of characters available for identification, with many features common to all known species of *Macrobrachium*, morphological identification of species of this genus is quite difficult (Qing‐Yi, Qi‐qun, & Wei‐bing, 2009). Characterization based only on morphological examination could lead to under‐ or overestimation of biodiversity (Lefébure, Douady, Gouy, & Gilbert, 2006). Given the current scenario, unbiased taxonomic classification through both morphological characterization and molecular characterization could shed more light into the diversity of this genus in the region.

Several studies have used mitochondrial DNA sequence data from the 16S rRNA and cytochrome c oxidase subunit 1 (CO1) genes to characterize Asian *Macrobrachium* taxonomy, biogeography, evolution, and life history (Liu, Cai, & Tzeng, 2007; Murphy & Austin, 2003, 2005; Pileggi & Mantelatto, 2010; Qing‐Yi et al., 2009; Vergamini, Pileggi, & Mantelatto, 2011). Microsatellites have also been developed for *Macrobrachium rosenbergii* De Man, 1879 (Divu, Khushiramani, Malathi, Karunasagar, & Karunasagar, 2008). The emergence of next‐generation sequencing tools has revolutionized taxonomic classification studies, as cost per sequencing output is continuously decreasing (Kilian et al., 2012). This has resulted in a shift of focus from molecular identification studies using universal genetic markers to high‐throughput genotyping using single nucleotide polymorphisms (SNPs). One of the emerging new genotyping technologies is Diversity Arrays Technology (DArT) (Imelfort, Batley, Grimmond, & Edwards, 2009; Kilian et al., 2012), which allows for simultaneous detection of several thousand of DNA polymorphisms (depending on the species) by scoring the presence or absence of DNA fragments in genomic representations generated from genomic DNA through a process of complexity reduction (Kilian et al., 2012). The efficacy of DArT markers in the analysis of genetic diversity, population structure, association mapping, and construction of linkage maps has been demonstrated for a variety of species (Appleby, Edwards, & Batley, 2009). DArT does not rely on previous sequence information for initial marker development, and this makes it the chosen platform for genetic characterization of species with little sequence information like African *Macrobrachium* (Sánchez‐Sevilla et al., 2015).

This study sought to determine the morphological and genetic diversity of *Macrobrachium* species in the main rivers of the South, South West, and Littoral regions of Cameroon using Konan (2009) key and DArT technology. It will serve to validate the current morphological‐based classification of West Africa *Macrobrachium* and contribute to the design of *Macrobrachium* breeding in Africa.

## 2 MATERIALS AND METHODS

### 2.1 Ethics statement

In Cameroon, freshwater prawn fishing is artisanal and an authorized activity. We bought fresh specimens from fishermen who chill and market wild prawn immediately after capture.

### 2.2 Sampling and collection of biological materials

**Figure 1:**
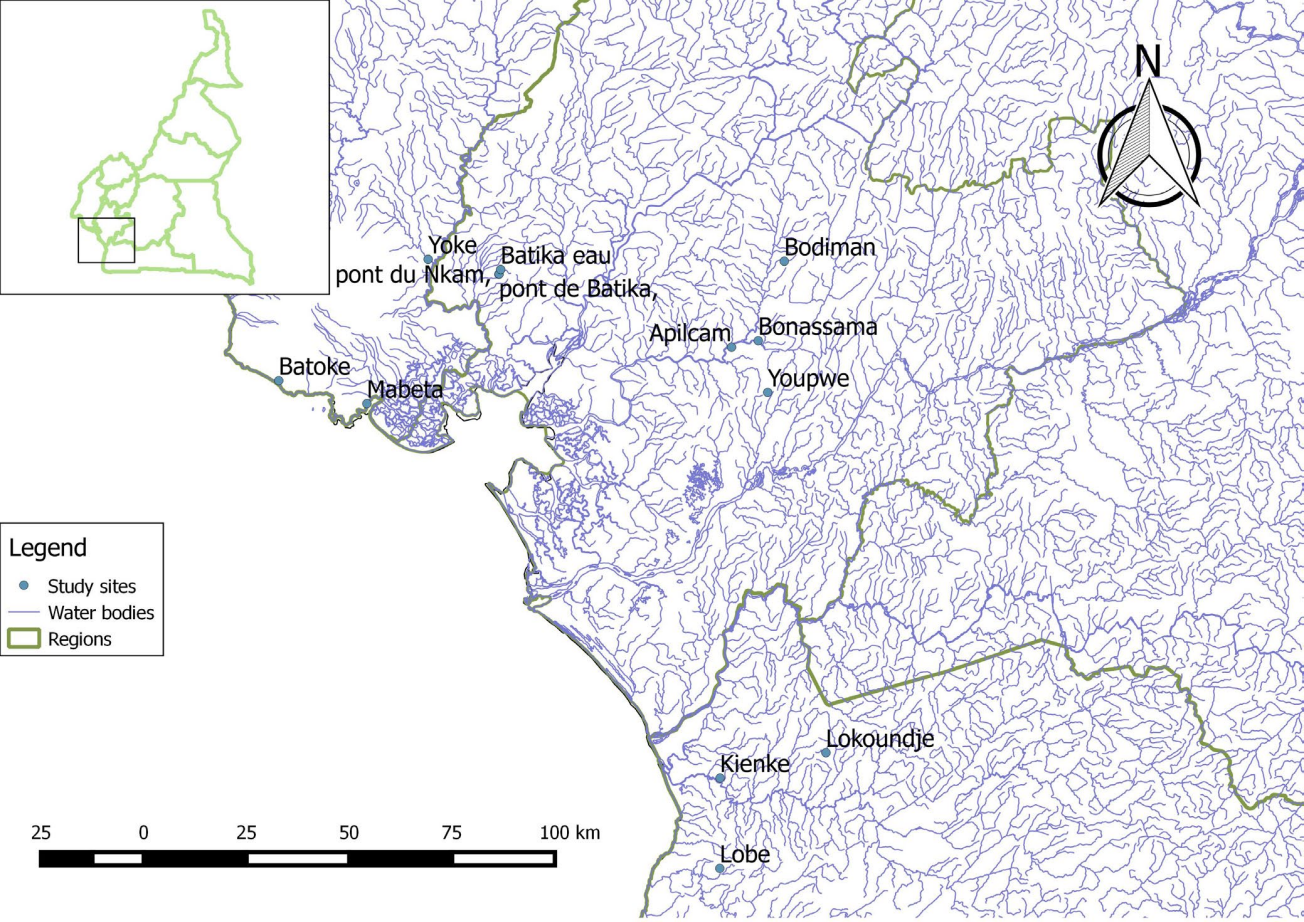
Map of the Atlantic Coast of Cameroon, showing the study sites

Between May 2015 and April 2016, *Macrobrachium* samples were collected monthly from fishermen catches at Lokoundje, Kienke, and Lobe rivers, in the South region; at Batoke, Mabeta, and Yoke rivers in the South West region; and at Nkam and Wouri rivers in the Littoral region, Cameroon. Coordinates of each collection point were taken using GPS (Figure 1). Samples were transported to the laboratory of the Institute of Agricultural Research for Development (IRAD) Batoke, Limbe, for measurements and taxonomic examinations.

### 2.3 Morphological identification of prawns

Before measurements, specimens were weighed individually using an electronic balance, coded, and preserved in 95% ethanol. Morphometric variables were recorded according to the measurement technique described by Kuris, Ra'anan, Sagi, and Cohen (1987) for the separation of morphotype of *M. rosenbergii*. Measurements of all characters were made to the nearest 0.01 mm using dial calipers type Stainless Hardened (range 0–200 mm) for the measurement of large specimens, and with magnifying binocular glasses for small specimens. All dimensions of the two legs of the second pair of the pereiopods and their joints were taken along the external lateral line. For each of the specimens collected, a total of 33 morphometric and six meristic characters were recorded (Appendix 1). After measurement, the specimens were identified to species level using the key described by Konan (2009) (Appendix 2). The Monod (1980) key was used when the species description was not found in Konan key. Samples were then stored in 95% ethanol for further molecular analysis.

### 2.4 Measurements of physicochemical parameters

Measurements of water physicochemical parameters of the rivers were done according to APHA (1998) and Rodier, Legube, and Brunet (2009) standards to see whether they have an influence in the distribution of *Macrobrachium* species in the three regions. Water temperature and dissolved oxygen were monitored monthly using oxygen meter (HI 9146, Hanna, Italy), while pH was measured using a pH meter (HI 98129, Hanna, USA).

### 2.5 Morphometric analysis

The Hierarchical Ascending Classification (AHC) based on Euclidean distance and Ward's algorithm was carried out to cluster species identified according to their morphometric similarities.

### 2.6 DNA extraction and genotyping

Due to financial limitations, a smaller set of 94 samples out of 1,566 collected (Appendix 3) was selected for molecular analysis. These samples were selected purposely (a) to represent all the species identified in the morphological analysis and (b) be a representative of sampled rivers and regions in order to assess potential genetic substructure among regions. Total genomic DNA was extracted from the muscle tissue of a pleopod using the DNeasy Blood/Tissue Kit (Qiagen, Germany), according to the manufacturer's instructions. Subsequently, 30 µl of 50–100 ng/µl for each sample was sent to Diversity Arrays Technology Pty Ltd. (DArT P/L) (http://www.diversityarrays.com/dart-mapsequences), for genotyping using a Genotyping‐by‐sequencing (GBS) approach as described by Elshire et al. (2011) using 52,834 DArT markers.

A total of 93 samples were successfully genotyped comprising 18 samples from *M. dux*; 18, *M. macrobrachion*; 18, *M. sollaudii*; 17, *M. vollenhovenii*; 12, *M. chevalieri*; 5, *M. felicinum*; and 5, *M*.* *sp.

### 2.7 Data filtering

Genotypic data quality control and checks were undertaken using PLINK v 1.9 (Purcell et al., 2007) entailing removal of SNPs with <80% call rate and <5% minor allele frequency (MAF). Consequently, a total of 1,814 SNPs were remained for further analysis.

### 2.8 Genetic diversity

Minor allele frequencies (MAF) were estimated using PLINK v 1.9 (Purcell et al., 2007). The distribution of MAF in each species was represented as the proportion of all the SNPs used in the analysis and subsequently grouped into five classes: [0.0,0.1], [0.1,0.2], [0.2,0.3], [0.3,0.4], and [0.4,0.5]. The proportions of SNPs in each class were then graphed for comparison between species using R (R Core Team, 2017).

Observed and expected heterozygosities were calculated using ARLEQUIN software, version 3.5 (Excoffier & Lischer, 2010). The expected heterozygosity per locus was calculated as follows:

$$\hat{H}=\frac{n}{n-1}\left(1-\sum_{i=1}^{k}p_{i}^{2}\right)$$

where *n* is the number of gene copies in the sample, *k* is the number of haplotypes, and *p_i_* is the sample frequency of the *i*th haplotype.

### 2.9 Population structure

Principal component analysis (PCA) was performed using PLINK (Purcell et al., 2007) and results were visualized using the GENESIS package (Buchmann & Hazelhurst, 2015) in R v 3.4.4.

A model‐based unsupervised clustering method implemented in the program ADMIXTURE v. 1.3.0 (Alexander, Novembre, & Lange, 2009) was used to estimate the genetic composition of individual prawns using the 1,814 markers. The analysis was run with *K* (number of distinct species) independent runs ranging from 2 to 20. A 10‐fold cross‐validation (CV = 10) was specified, with the resultant error profile used to explore the most probable number of clusters (*K*), as described by Alexander et al. (2009). The optimal *K* was confirmed using discriminate principal component analysis (DPCA) and the Evanno Δ*K* methods. Graphical display of the admixture analysis was done using the Microsoft Excel package.

### 2.10 Analysis of genetic relationships

Pairwise *F*_ST_ was computed with 1,000 permutations using ARLEQUIN software, version 3.5 (Excoffier & Lischer, 2010). A phylogenetic tree was then generated from a matrix of pairwise *F*_ST_ estimates using Splits Tree software, version 4.13.1 (Huson & Bryant, 2006).

## 3 RESULTS

### 3.1 Physicochemical parameters of the rivers

The physicochemical parameters of the eight rivers sampled are shown in Table 1. The mean pH of all the rivers was between 7.08 and 7.70. Temperature varied from 23.66 to 29.28°C. Lokoundje River recorded the highest mean temperature (26.64°C). Dissolved oxygen was highly variable in the rivers of the Littoral region (Nkam River: 2–8 mg/L), whereas in the South West region, it was high in all the rivers with the lowest value recorded in Batoke River (5–6.63 mg/L).

**Table 1:** Physicochemical parameters measured in eight rivers of the coastal area of Cameroon

Regions	Rivers	*T* (°C)	DO (mg/L)	pH
Littoral	Nkam			
	Mean	25.68	6.5	7.2
	*SD*	0.58	1.52	0.58
	Range	24.6–26.8	2.0–8.0	6.2–8.6
	Wouri			
	Mean	25.91	5.8	7.08
	*SD*	0.61	1.62	0.58
	Range	24.7–27	3.5–8.0	6.1–8.01
South	Kienke			
	Mean	25.51	4.83	7.18
	*SD*	1.33	0.51	0.11
	Range	23.66–28.32	4.1–5.68	6.61–7.23
	Lobe			
	Mean	25.63	4.29	7.1
	*SD*	1.29	0.31	0.15
	Range	23.51–28.71	4–5.2	6.6–7.25
	Lokoundje			
	Mean	26.64	6.42	7.31
	*SD*	1.49	0.71	0.17
	Range	24.6–29.28	5.5–7.7	6.9–7.7
South West	Batoke			
	Mean	25.5	5.67	7.7
	*SD*	0.62	0.9	0.53
	Range	24.1–26.5	5–6.63	6.5–8.8
	Mabeta			
	Mean	24.71	6.80	7.28
	*SD*	0.35	0.87	0.44
	Range	24.1–26.7	5.90–7.99	6.1– 8.4
	Yoke			
	Mean	25.5	6.31	7.3
	*SD*	0.88	0.42	0.35
	Range	24.3–27	5.91–7.6	6.5–8.0

### 3.2 Morphological analysis and distribution of the species in the three regions

**Figure 2:**
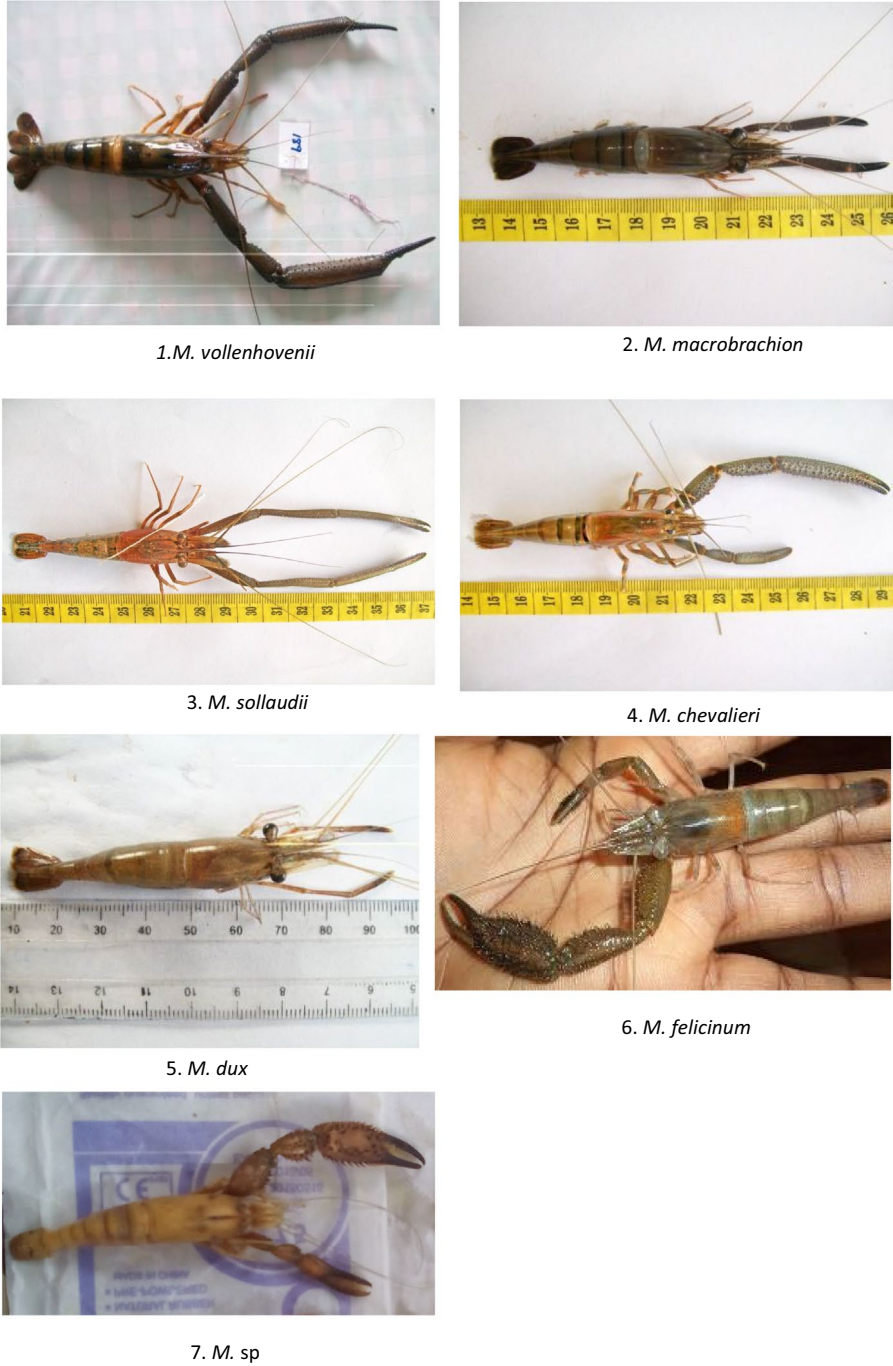
Images of the seven species of *Macrobrachium* identified through morphological analysis in the coastal area of Cameroon. 1: *M. vollenhovenii*, 2: *M. macrobrachion*, 3: *M. sollaudii*, 4: *M. chevalieri*, 5: *M. dux*, 6: *M. felicinum*, 7: *M*.* *sp

Of the 1,566 specimens examined morphologically using Konan (2009) and Monod (1980) keys (Table 2), 916 (58.5%) were recorded in South region, 398 (25.5%) in South West region, and 252 (16.1%) in Littoral region. Based on the morphometric measures and species allocation criteria described by the keys, seven prawn species were identified. These were *M. vollenhovenii*, *M. macrobrachion*, *M. sollaudii*, *M. dux*, *M. chevalieri*, *M. felicinum*, and an undescribed species, *M*.* *sp (Figure 2). These species were not found in all the three regions (Table 3). *M. felicinum* was found only in the South region, *M*.* *sp was found exclusively in the South West region, while *M. chevalieri*, *M. felicinum*, and *M*.* *sp were absent in the Littoral region.

**Table 2:** Species and sample size and sampling regions of *Macrobrachium* spp. identified using morphological analysis

Region	Rivers	*M. chevalieri*	*M. dux*	*M. felicinum*	*M. macrobrachion*	*M. sollaudii*	*M*.* *sp	*M. vollenhovenii*	Total
South	Kienke	18	40	8	78	25		90	259
	Lobe	21	36	4	79	27		124	291
	Lokoundje	33	45	28	79	14		167	366
Littoral	Nkam		56			46			102
	Wouri		54		20	59		17	150
South West	Batoke	41	23		8	13	79	15	179
	Mabeta		10		18	19		27	74
	Yoke	5	3		42	2		93	145
Total		118	267	40	324	205	79	533	1,566

**Table 3:** Distribution of *Macrobrachium* in the three regions

Species	Littoral	South	South West
*M. vollenhovenii*	+	+	+
*M. macrobrachion*	+	+	+
*M. dux*	+	+	+
*M. sollaudii*	+	+	+
*M. chevalieri*	−	+	+
*M. felicinum*	−	+	−
*M*.* *sp	−	−	+

Note

Key: + = presence, − = absence.

### 3.3 Morphometric similarities between species identified

**Figure 3:**
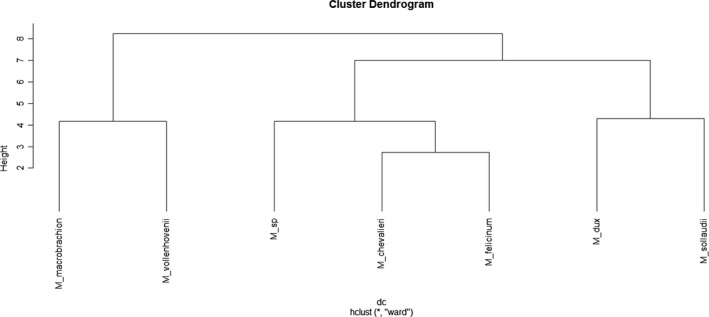
Dendrogram of hierarchical cluster analysis between species of *Macrobrachium* from coastal area of Cameroon

A dendrogram of hierarchical cluster analysis showing morphological similarities between *Macrobrachium* species is shown in Figure 3. The dendrogram shows the presence of three main branches (i.e., groups of species), the first one groups *M. vollenhovenii* and *M. macrobrachion*, the second one groups *M*.* *sp, *M. chevalieri* and *M. felicinum* with the latter two species being more closely related, and the third branch groups *M. dux* and *M. sollaudii*.

### 3.4 Genetic diversity

**Figure 4:**
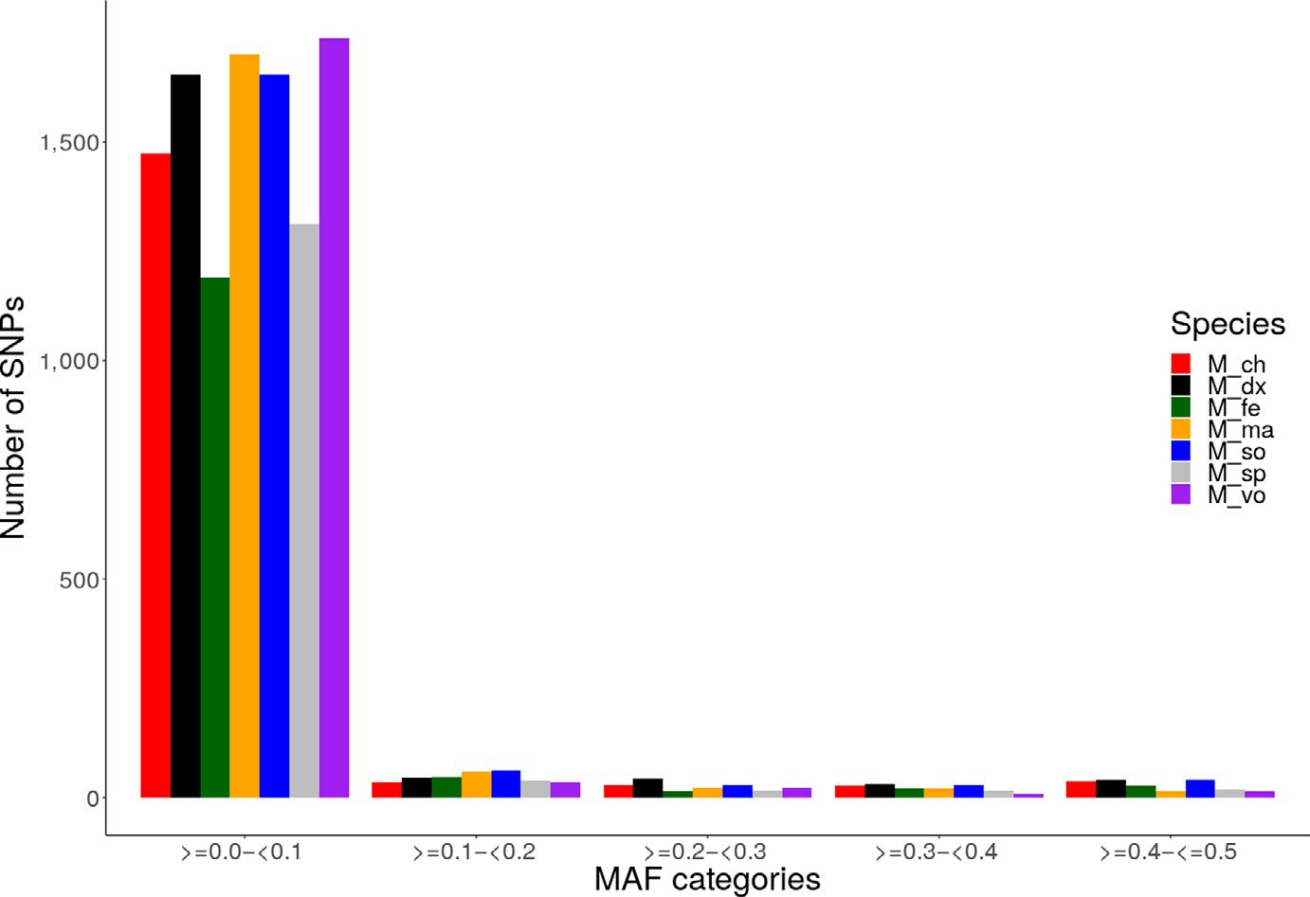
Minor allele frequency (MAF) distribution for each species. MAF were calculated for each species and SNPs binned into five categories (≥0 to 0.1, ≥0.1 to 0.2, >0.2 to <0.3, ≥0.3 to <0.4, and ≥0.4 to ≤0.5) based on their MAF. *M: Macrobrachium*, ch*: chevalieri;* dx*: dux;* fe*: felicinum;* ma*: macrobrachion;* so*: sollaudii;* vo*: vollenhovenii*

Diversity Arrays Technology markers presented an average genotype call rate of 40.8% and an average scoring reproducibility of 99.9%. The PIC values ranged from 0.02 to 0.50 with an average of 0.15. The heterozygosity estimates and minor allele distribution are presented in Table 4 and Figure 4, respectively. Approximately 85% of all loci had minor allele frequencies <0.1.

**Table 4:** Genetic diversity parameters of *Macrobrachium* from the coastal region of Cameroon. Values are estimates ± *SD*

Groups	Observed Het	Expected Het	Monomorphic loci	Polymorphic loci	*F*_IS_	*p*
*M*.* *sp	0.41 ± 0.32	0.36 ± 0.15	871	61	−0.34	.90
*M. dux*	0.31 ± 0.27	0.26 ± 0.17	821 ± 5	111 ± 5	−0.28	1
*M. macrobrachion*	0.05 ± 0.10	0.14 ± 0.09	731 ± 187	201 ± 187	0.61	.01
*M. chevalieri*	0.35 ± 0.29	0.31 ± 0.17	834 ± 0	98 ± 0	−0.19	.97
*M. sollaudii*	0.27 ± 0.25	0.24 ± 0.17	822 ± 5	110 ± 5	−0.18	.99
*M. vollenhovenii*	0.15 ± 0.14	0.20 ± 0.15	858 ± 7	74 ± 7	0.04	.20
*M. felicinum*	0.45 ± 0.32	0.37 ± 0.16	858	74	−0.32	.88

Note

Het: heterozygosity; *M: Macrobrachium*; *F*_IS_: inbreeding coefficient

### 3.5 Population structure

**Figure 5:**
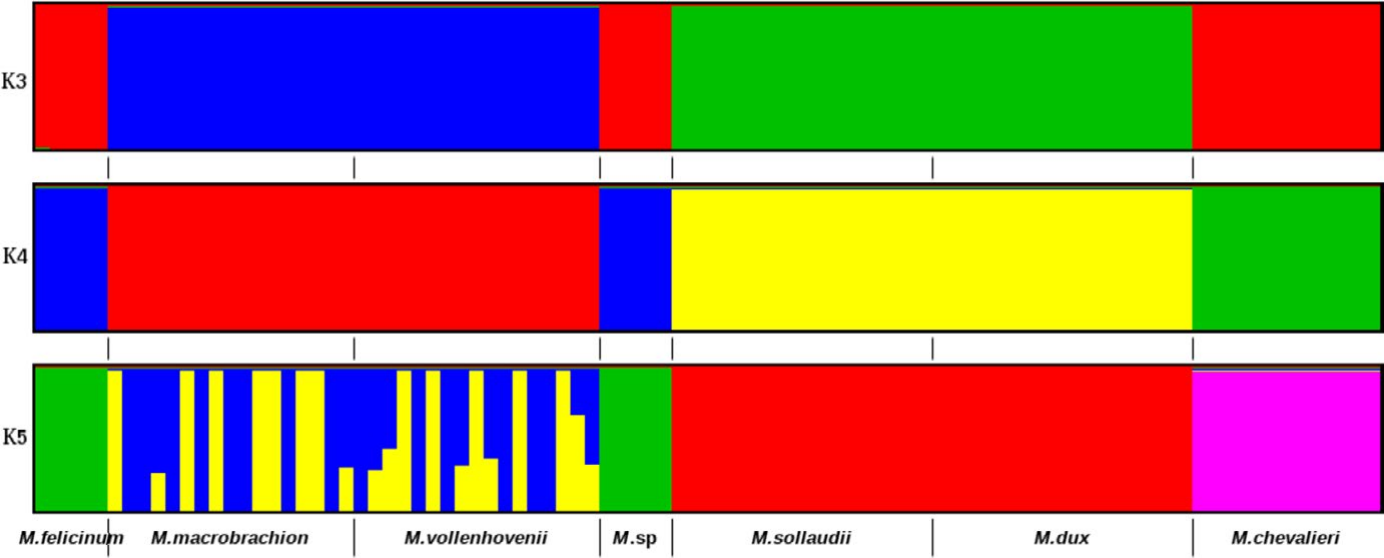
Admixture bar plot showing species proportions at assumed clusters *K* = 3–5

The admixture analysis revealed four main clusters (*K* = 4) (Figure 5). At *K* = 3, *M*.* *sp, *M. chevalieri*, and *M. felicinum* species clustered together as a single group, *M. dux* and *M. sollaudii* species clustered as a second group, while *M. macrobrachion* and *M. vollenhovenii* clustered as a third group. At *K* = 4, *M. chevalieri* formed a distinct group split from group 1. At *K* = 5, there was no further substructure that emerged. However, individuals in group 3 that consist of *M. macrobrachion* and *M. vollenhovenii* revealed substantial admixture derived from two hitherto distinct genetic backgrounds.

**Figure 6:**
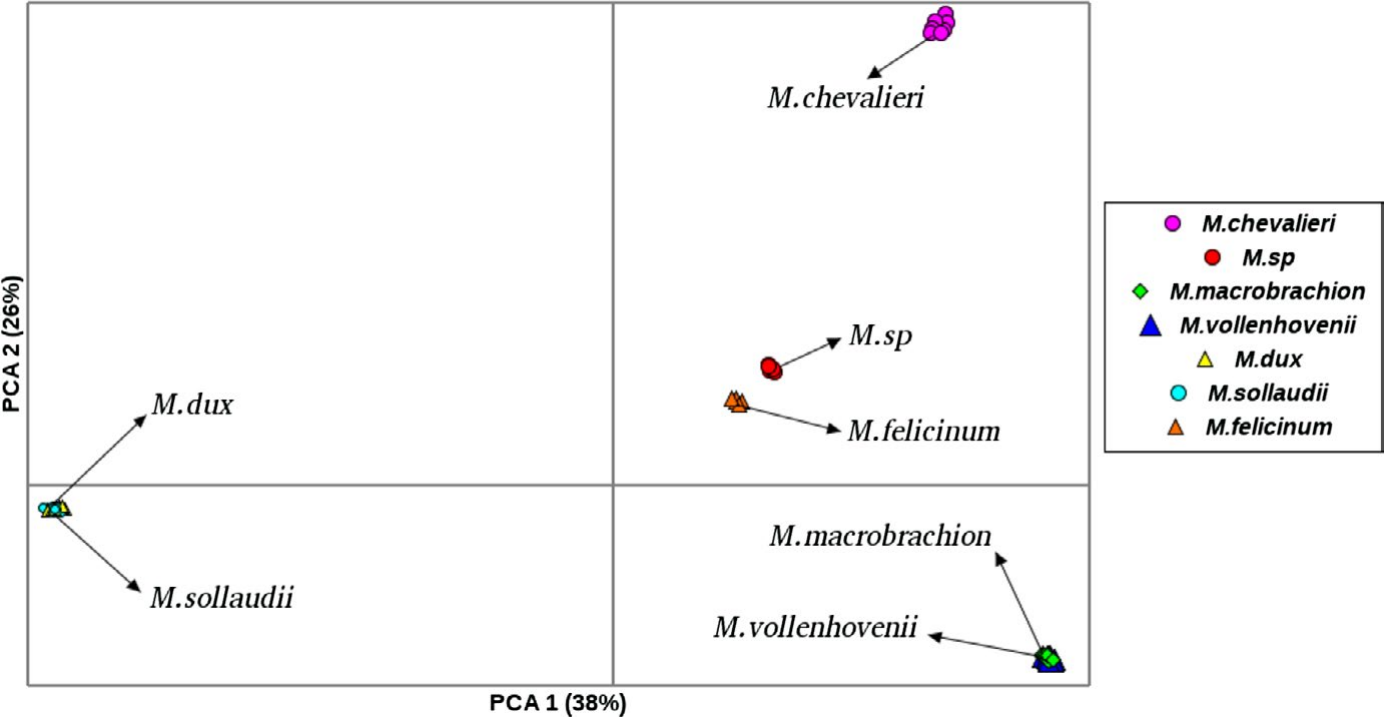
Principal component analysis (PCA) plot of *Macrobrachium* based on 1,814 DArT markers

Results from PCA revealed five clusters as shown in Figure 6. The first principal component accounted for 38% of the total variation and separated *M. dux* and *M. sollaudii* from the rest of the species. The second component accounted for 26% of the total variation and separated *M. vollenhovenii* and *M. macrobrachion* from the other species. *M*.* *sp and *M. felicinum* species formed two distinct groups that were in close proximity, while *M. chevalieri* formed a distinct cluster.

### 3.6 Population differentiation

The genetic distance of the species based on *F*_st_ ranged from −0.0105 to 0.9461 (Table 5). The lowest genetic distance (*F*_st_ *=* −0.0105) was observed between *M. dux* and *M. sollaudii*, while the highest differentiation (*F*_st_ = 0.9461) was obtained between *M*.* *sp and *M. vollenhovenii*.

**Table 5:** Pairwise *F*_st_ among *Macrobrachium* species

	*M*.* *sp	*M. dux*	*M. macro*	*M. che*	*M. sol*	*M. vol*	*M. fel*
*M*.* *sp	0						
*M. dux*	0.9268	0					
*M. macro*	0.8647	0.8889	0				
*M. che*	0.9339	0.9280	0.8604	0			
*M. sol*	0.9265	−0.0105	0.8887	0.9279	0		
*M. vol*	0.9461	0.9360	0.0139	0.9327	0.9360	0	
*M. fel*	0.9077	0.9195	0.8549	0.9262	0.9194	0.9402	0

Abbreviations: *che*, *chevalieri*; *fel*, *felicinum*; *M*, *Macrobrachium*; *macro*, *macrobrachion*; *sol*, *sollaudii*; *vol*, *vollenhovenii*.

**Figure 7:**
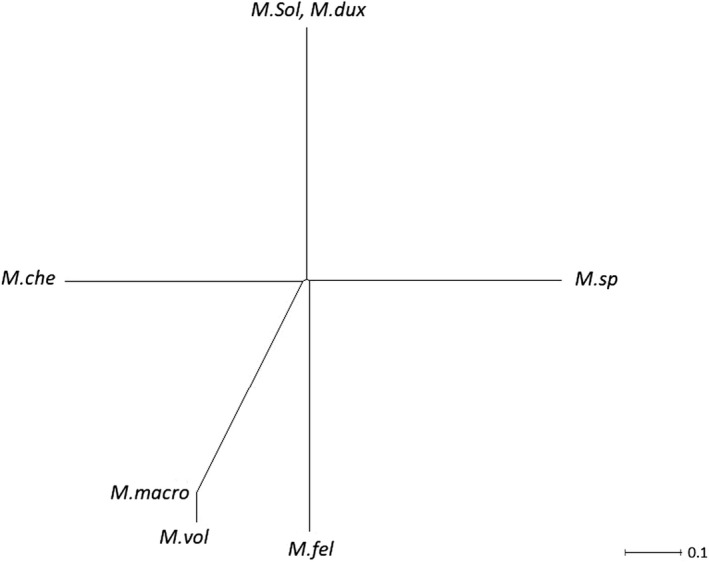
Phylogenetic tree of *Macrobrachium* species detected in the study. *che*, *chevalieri*; *fel*, *felicinum*; *M*, *Macrobrachium*; *macro*, *macrobrachion*; *sol*, *sollaudii*; *vol*, *vollenhovenii*

In line with the PCA findings, the phylogenetic tree differentiated *M*.* *sp from *M. felicinum* (Figure 7). More interestingly, *M. dux* and *M. sollaudii* appeared at the same node.

## 4 DISCUSSION

Major systematic treatments of freshwater prawns have been based on morphological characteristics alone (Murphy & Austin, 2003; Rossi & Mantelatto, 2013). The complexity of prawns of the genus *Macrobrachium* where morphological traits have been shown to be strongly influenced by the environment and may not be indicative of underlying genetic divergence (Dimmock, Willamson, & Mather, 2004) has often led to over/underestimation of the diversity (Lefébure et al., 2006). This study sought to use both morphological and genetic approaches in a bid to not only correctly identify the prawn species present in Cameroon, but also contrast the allocation of individual prawns to prospective species using a combined and more reliable analysis.

Based on the morphological key, the samples obtained represent seven distinct species in the coastal area of Cameroon, namely *M. vollenhovenii*, *M. macrobrachion*, *M. sollaudii*, *M. dux*, *M. chevalieri*, *M. felicinum*, and an undescribed species *M*.* *sp. This is in contrast with previous studies on known *Macrobrachium* in Cameroon, where four (Monod, 1980) and six (Makombu et al., 2015) species were identified. The difference in the number of identified species could be explained by the sampling strategy. The Makombu et al. (2015) study was limited to the South region, while the Monod (1980) focused on general investigation of *Macrobrachium* in West Africa with limited sampling in Cameroon. Additionally, the Monod (1980) study was undertaken in a short period of time with no information of the rivers and regions where the specimens were collected. The present study took into consideration eight main rivers of the three regions that rim the coastal area, coupled with 1 year of data collection.

There was differential distribution of species across the three regions. The Littoral region had the least species abundance with only four species sampled, all of which were also present in the South West and South regions. South West and South region recorded the same number of species (6) with the difference that *M. felicinum* was found only in the South region and the undescribed species *M*.* *sp found only in one river (Batoke River) in the South West region. The absence of *M*.* *sp in two other rivers of the same region (Yoke and Mabeta rivers) could be due to the relatively high dissolved oxygen recorded in these two rivers. It may also be a habitat selection for *M*.* *sp. Given that *M*.* *sp has been identified for the first time in Cameroon, further studies on its biology and ecology are highly recommended.

The relationship between the species identified in this study based on morphological features is the same as that observed in the Makombu et al. (2015) and Konan (2009) studies. The only difference is the identification of a new species, christened in this study as *M*. sp. The concordance between this phenotypic relationship and the genetic relationship based on DNA analysis served as the basis of this study. This is important given the influence that environmental factors have on morphological characteristics. It is possible that similar ecotypes sourced from different regions could be identified differently. At the genetic level, DArT markers used in the present study displayed fairly low polymorphism information content (average PIC = 0.15). These low values of PIC deviate from those seen in other commercially important nonaquatic species (PIC values range between 0.30 and 0.44; Raman et al., 2010; Sánchez‐Sevilla et al., 2015; Wenzl et al., 2004). The use of DArT in characterization of animals (and particularly aquatic animals) has been limited (Melville et al., 2017). In this study, even though up to 50,000 SNP markers were available after genotyping, only 1,814 met the criterion for further analysis, which limits the extent of the genetic diversity that can be captured. A larger study with more robust markers is paramount to completely characterize the genetic structure and relationships among the target species.

The low genetic distance between *M. dux* and *M. sollaudii*, indicated from *F*_ST_ values, PCA clustering, and admixture results indicate very high genetic similarity between them. Whereas in this study we do not have conclusive evidence to suggest they are the same species, at the genetic level they seem to be highly similar. The phenotypic differences seen between them may be due to differential expression of genes that control the morphometric features used for classification (Dimmock et al., 2004). The phenotypic differences observed may also be the possibility of morphotypes within a species. *M. sollaudii* male has 2nd pereiopods (chelipeds) more developed than *M. dux* male, this could be two male morphotypes of a same species. Moreover, looking at the sex ratio, male highly dominate female in *M. sollaudii* collected in the three regions (>90% male). Cases of morphotypes within the genus *Macrobrachium* have been documented. Kuris et al. (1987) reported three male morphotypes of *M. rosenbergii*: the dominant blue clawed males (BC), the subordinate orange clawed males (OC), and the nonterritorial small clawed males (SM). Wortham and Maurik (2012) also reported three morphotypes within *M. grandimanus* (Randall, 1840): females, small symmetrical males, and large asymmetrical males. Study on morphotypic differentiation of species of this group of prawn is highly recommended.

Similarly, the results from this study indicate that *M*. *macrobranchion* and *M. vollenhovenii* are highly related and could represent panmictic populations. The admixture results at *K* = 5 allude to two distinct genetic stocks that exhibit the possibility of interbreeding and extensive gene flow to give rise to admixed individuals. The colocation of these species in the same rivers and habitat, as well as their amphidromous behavior patterns characterized by female migration from rivers to estuaries following hatching, larval development in saltwater, and a return upriver migration by postlarvae (Bauer and Delahoussaye, 2008), possibly provides ample opportunity for mating and hence gene flow between these two species. The lack of genetic differentiation between these species has been previously observed. J. G. Makombu et al. (unpublished results) observed similar results using mitochondrial DNA, increasing the possibility that they are descended from the same maternal genetic stock. Konan (2009) also found no genetic differentiation between *M. vollenhovenii* and *M. macrobrachion* using enzymatic polymorphism. However, given the quite divergent marker profiles observed, there is evidence to suggest that these species are different. The large differences in number of polymorphic markers and genetic heterozygosity measures observed point to significant differentiation driven by differential speciation. The fact that they are colocated in the same rivers and habitats rules our differential manifestation of environmental influences. Perhaps a study of differential gene expression may shed more light as to the genetic basis of the huge phenotypic differences. It is instructive to note that during morphological analyses, specimens having characteristics of both of *M. vollenhovenii* and *M. macrobrachion* were found. These “hybrid” individuals may represent the admixed individuals borne out of the two species. This could not be further investigated owing to limited resources available for this study. Given the relatively small marker set used, a deeper characterization of these species using dense genetic markers (both organellar and autosomal) would be necessary to remove any doubts as to the genetic relationship between them.

In contrast to the admixture results, both the PCA and the genetic distance estimates visualized by the phylogenetic tree separated *M*.* *sp from *M. felicinum*. The lack of differentiation based on genetic admixture could be because of small number of samples used for genotyping (*M*.* *sp: *N* = 5; *M. felicinum*: *N* = 5), which would limit differences in allelic patterns observable. Both species have low relative abundance; hence, the lower number of samples is obtained. Despite their close similarity in terms of phenotypic and morphometric features, they are not located in the same habitat; hence, there is reasonable chance to conclude that they are different species.

According to Dimmock et al. (2004), *Macrobrachium* is a notoriously difficult genus taxonomically, as the morphological plasticity of important traits changes extensively and gradually during the growth and is influenced by environmental parameters. Morphologically similar species are often quite genetically distinct. Analogous situations have been reported for some marine crustaceans (Knowlton, 2000) and freshwater macroinvertebrates (Baker, Hughes, Dean, & Bunn, 2004; Shih, Ng, & Chang, 2004).

The perils of morphological taxonomy of species of the genus *Macrobrachium* have been recorded in previous studies (Murphy & Austin, 2005; Vergamini et al., 2011). So far, many studies have called into question morphological classification of members of this group (Boulton & Knott, 1984; Fincham, 1987; Holthuis, 1952). Additionally, Qing‐Yi et al. (2009), Murphy and Austin (2002, 2004), and Short (2004) invalidated current morphologically based classification of Asian *Macrobrachium* species. Holthuis (1952) listed a number of reasons why classification of this genus is very difficult. These include a restricted number of characters available for identification, with many features common to all species, sexual dimorphism, and some species possibly being sexually mature before all body parts are fully developed. Use of molecular markers allows us to detect the genetic uniqueness of a particular individual, species, or population irrespective of the challenges enumerated above (Maralit & Santos, 2015).

## 5 CONCLUSION

This study has demonstrated that the use of morphological parameters for the classification of *Macrobrachium* species of the coastal area of Cameroon is fraught with possible misclassification especially for species that are panmictic with high gene flow. Genetic characterization has confirmed that *M. chevalieri* is a genetically different species from *M*.* *sp and *M. felicinum* despite morphological similarity. Additionally, *M. vollenhovenii* and *M. macrobrachion* display great gene flow between two genetic backgrounds, likely as a result of a panmictic population undergoing localized divergence, while *M. dux* and *M. sollaudii* seem to be conspecific. However, the results obtained in this study were limited by the low average PIC value and call rate of DArT markers used, coupled with the small number of individual used for some species. This study constitutes a critical first step in developing a genetic test for accurate identification of *Macrobrachium* species of coastal Cameroon.

## CONFLICT OF INTEREST

Evans K. Cherulyot and Fidalis D. N. Mujibi were employed by company USOMI, and Eric Mialhe was employed by company Concepto Azul. All other authors declare no competing interests.

## AUTHOR CONTRIBUTIONS

JM, FS, FM, EM, and PO conceived and coordinated the work. JM, ET, PZ, AE, AN, and BO acquired data. FM, FS, EC, GT, OS, ET, and JN analyzed and interpreted the data. JM drafted the manuscript. FS, FM, GT, and OS contributed to revisions and edits of the manuscript.

## Data Availability

Link for the data: GPS points: https://figshare.com/s/08de4b966929dfd56881; Morphological data: https://figshare.com/s/827a37559c806e7c11b6; Molecular data: https://figshare.com/s/3af1e859f4622ea27f1d.

## APPENDIX 1

### Detailed description of morphometric and meristic characters used in this study

**Table:**

No.	Characters	Abbreviation	Description
Morphometric			
1	Total length	TL	Distance between rostrum tip and the distal tip of the telson with shrimp stretched out
2	Carapace length	CL	Distance between the posterior margin of the right orbit and the midpoint of the posterior margin of the carapace
3	Rostrum length	R	Distance of epigastric tooth basis to rostrum tip
4	Head length	H	Distance between the rostrum tip and the midpoint of the posterior margin of the carapace
5	Telson length	Te	Distance of posterior margin of sixth abdominal somite to telson tip
6	Telson width	Tew	Distance between lateral margin of telson taken in his basis
7	Carapace width	Cw	Distance between lateral margin of cephalothorax
8	Carapace height	CH	Distance between dorsal and ventral end of carapace
9 & 10	Pereiopod length	L1 & L2	Distance between the proximal margin of the ischium and the distal tip of propodus
11 & 12	Ischium length	I1 & I2	Distance from the proximal to the distal end of ischium
13 & 14	Merus length	M1 & M2	Distance between proximal and distal margin of merus
15 & 16	Carpus length	C1 & C2	Distance from the proximal to the distal end of carpus
17 & 18	Palm length	P1 & P2	Distance between proximal and distal margin of palm
19 & 20	Dactylus length	D1 & D2	Distance between proximal and distal margin of dactyl
21 & 22	Distal tooth‐fixed digit tip	Df1 & Df2	Distance from distal tooth of fixed digit to propodus tip
23 & 24	Distal tooth‐dactylus tip	Dt1 & Dt2	Distance from distal tooth of dactylus tip
25 & 26	Ischium width	Iw1 & Iw2	Distance between lateral line of ischium
27 & 28	Merus width	Mw1 & Mw2	Distance between lateral line of merus
29 & 30	Carpus width	Caw1& Caw2	Distance between lateral line of carpus
31 & 32	Palm width	Pw1 & Pw2	Distance between lateral line of palm
33	Eye diameter	ED	Distance between lateral lines of orbit taken on right eye
Meristics			
1	Dorsal teeth	Dr	Teeth number on rostrum dorsal line
2	Ventral teeth	Vr	Teeth number on rostrum ventral line
3	Postorbital teeth	Po	Number of rostrum postorbital teeth
4	Spines of telson	St	Spines number on telson dorsal face
5	Spine of palm	Sp	Spines number on interne lateral line of palm
6	Dactylus teeth	Dt	Number of dactylus teeth

## APPENDIX 2

### Identification keys of *Macrobrachium*

- Key to family and genus (Monod, 1980)

Legs of the first two pairs of pereiopods different and finished by pincers; legs of the second pair are well developed and end with strong pincers; rostrum is developed and has teeth on both sides; medium to large or very large size (45–182 mm) …… Palaemonidae (*Macrobrachium*).

- Key to species of Macrobrachium according to Konan, 2009

1. The carpus of the second pereiopod is shorter or same length with the merus, the ratio of the length of carpus/length of merus is <1.08 …… 2

The carpus of the second pereiopod is longer than the merus; the ratio of the length of carpus/length of merus is >1.08 …… 3

1. The merus is 0.20–0.27 times longer than large; the length of the carpus is 0.70–0.95 times the length of the palm; the rostrum tip is at the same level or longer than the antenna scale …… *M. macrobrachion*

The merus is 0.27–0.47 times longer than large; the length of the carpus is 0.45–0.75 times the length of the palm; the rostrum tip is shorter than the extremity of the antenna scale …… *M. vollenhovenii*

1. The carpus is longer than the palm (length of carpus/length of palm = 1.17–1.39); the merus is as long or longer than the palm (M/P1 = 0.98–1.14) …… 4

The carpus is shorter or the same length with the palm (length of carpus/length of palm = 0.61–1); the merus is shorter than the palm (M/P1 = 0.58–0.90) …… 5

1. The merus length is greater than the palm length (M/P1 = 1.02–1.06); the carpus length is greater than the carapace length (103.40%–149.91% Carapace length); the length of the ischium/length of merus is <2/3 …… *M. sollaudii*

The merus length and palm length are almost the same (M/P1 = 0.98–0.99); the carpus length is less than the carapace length (61.13%–95.62% carapace length); the length of the ischium/the length of merus is >2/3 …… *M. dux*

1. Fingers not bent, but they touch each other throughout the entire length and do not possess internal fur …… *M. thysi*

Fingers bent, do not touch each other and possess internal fur …… *M. felicinum*

## APPENDIX 3

### Collection site of 94 *Macrobrachium* samples

**Table:**

Sample number	Species	Sex	Rivers	Region
1	*Macrobrachium* sp	Male	Batoke	South West
2	*Macrobrachium* sp	Male	Batoke	South West
3	*Macrobrachium* sp	Male	Batoke	South West
4	*Macrobrachium* sp	Female	Batoke	South West
5	*Macrobrachium* sp	Female	Batoke	South West
6	*Macrobrachium dux*	Male	Batoke	South West
7	*Macrobrachium dux*	Female	Batoke	South West
8	*Macrobrachium dux*	Female	Yoke	South West
9	*Macrobrachium dux*	Male	Yoke	South West
10	*Macrobrachium dux*	Female	Mabeta	South West
11	*Macrobrachium dux*	Male	Mabeta	South West
12	*Macrobrachium macrobrachion*	Female	Mabeta	South West
13	*Macrobrachium macrobrachion*	Male	Mabeta	South West
14	*Macrobrachium macrobrachion*	Male	Mabeta	South West
15	*Macrobrachium macrobrachion*	Female	yoke	South West
16	*Macrobrachium macrobrachion*	Female	Yoke	South West
17	*Macrobrachium macrobrachion*	Female	Yoke	South West
18	*Macrobrachium vollenhovenii*	Male	Batoke	South West
19	*Macrobrachium vollenhovenii*	Male	Yoke	South West
20	*Macrobrachium vollenhovenii*	Female	Yoke	South West
21	*Macrobrachium vollenhovenii*	Male	Yoke	South West
22	*Macrobrachium vollenhovenii*	Male	Mabeta	South West
23	*Macrobrachium vollenhovenii*	Female	Mabeta	South West
24	*Macrobrachium chevalieri*	Female	Batoke	South West
25	*Macrobrachium chevalieri*	Female	Batoke	South West
26	*Macrobrachium chevalieri*	Female	Batoke	South West
27	*Macrobrachium chevalieri*	Male	Batoke	South West
28	*Macrobrachium chevalieri*	Male	Batoke	South West
29	*Macrobrachium chevalieri*	Male	Batoke	South West
30	*Macrobrachium sollaudii*	Male	Yoke	South West
31	*Macrobrachium sollaudii*	Male	Yoke	South West
32	*Macrobrachium sollaudii*	Male	Mabeta	South West
33	*Macrobrachium sollaudii*	Female	Mabeta	South West
34	*Macrobrachium sollaudii*	Male	Batoke	South West
35	*Macrobrachium sollaudii*	Male	Batoke	South West
36	*Macrobrachium dux*	Female	Nkam	Littoral
37	*Macrobrachium dux*	Male	Nkam	Littoral
38	*Macrobrachium dux*	Female	Wouri	Littoral
39	*Macrobrachium dux*	Female	Wouri	Littoral
40	*Macrobrachium dux*	Male	Wouri	Littoral
41	*Macrobrachium dux*	Male	Wouri	Littoral
42	*Macrobrachium vollenhovenii*	Female	Wouri	Littoral
43	*Macrobrachium vollenhovenii*	Male	Wouri	Littoral
44	*Macrobrachium vollenhovenii*	Male	Wouri	Littoral
45	*Macrobrachium vollenhovenii*	Male	Wouri	Littoral
46	*Macrobrachium vollenhovenii*	Female	Wouri	Littoral
47	*Macrobrachium macrobrachion*	Female	Wouri	Littoral
48	*Macrobrachium macrobrachion*	Female	Wouri	Littoral
49	*Macrobrachium macrobrachion*	Female	Wouri	Littoral
50	*Macrobrachium macrobrachion*	Male	Wouri	Littoral
51	*Macrobrachium macrobrachion*	Male	Wouri	Littoral
52	*Macrobrachium macrobrachion*	Male	Wouri	Littoral
53	*Macrobrachium sollaudii*	Male	Nkam	Littoral
54	*Macrobrachium sollaudii*	Male	Nkam	Littoral
55	*Macrobrachium sollaudii*	Male	Wouri	Littoral
56	*Macrobrachium sollaudii*	Female	Wouri	Littoral
57	*Macrobrachium sollaudii*	Male	Wouri	Littoral
58	*Macrobrachium sollaudii*	Male	Wouri	Littoral
59	*Macrobrachium chevalieri*	Female	Lobe	South
60	*Macrobrachium chevalieri*	Female	Lobe	South
61	*Macrobrachium chevalieri*	Male	Lokoundje	South
62	*Macrobrachium chevalieri*	Female	Lokoundje	South
63	*Macrobrachium chevalieri*	Female	Kienke	South
64	*Macrobrachium chevalieri*	Male	Kienke	South
65	*Macrobrachium felicinum*	Female	Lokoundje	South
66	*Macrobrachium felicinum*	Female	Lokoundje	South
67	*Macrobrachium felicinum*	Male	Lokoundje	South
68	*Macrobrachium felicinum*	Female	Lobe	South
69	*Macrobrachium felicinum*	Male	Lobe	South
70	*Macrobrachium felicinum*	Male	Kienke	South
71	*Macrobrachium macrobrachion*	Female	Lokoundje	South
72	*Macrobrachium macrobrachion*	Male	Lokoundje	South
73	*Macrobrachium macrobrachion*	Male	Lobe	South
74	*Macrobrachium macrobrachion*	Female	Lobe	South
75	*Macrobrachium macrobrachion*	Female	Kienke	South
76	*Macrobrachium macrobrachion*	Male	Kienke	South
77	*Macrobrachium vollenhovenii*	Female	Kienke	South
78	*Macrobrachium vollenhovenii*	Male	Kienke	South
79	*Macrobrachium vollenhovenii*	Male	Lokoundje	South
80	*Macrobrachium vollenhovenii*	Female	Lokoundje	South
81	*Macrobrachium vollenhovenii*	Male	Lobe	South
82	*Macrobrachium vollenhovenii*	Female	Lobe	South
83	*Macrobrachium sollaudii*	Male	Kienke	South
84	*Macrobrachium sollaudii*	Male	Lobe	South
85	*Macrobrachium sollaudii*	Male	Lokoundje	South
86	*Macrobrachium sollaudii*	Female	Lokoundje	South
87	*Macrobrachium sollaudii*	Male	Lobe	South
88	*Macrobrachium sollaudii*	Female	Kienke	South
89	*Macrobrachium dux*	Female	Lobe	South
90	*Macrobrachium dux*	Female	Kienke	South
91	*Macrobrachium dux*	Male	Kienke	South
92	*Macrobrachium dux*	Male	Lokoundje	South
93	*Macrobrachium dux*	Female	Lokoundje	South
94	*Macrobrachium dux*	Male	Lobe	South
